# Incidents related to the duration of medical devices in Intensive Care: a cross-sectional study

**DOI:** 10.1590/0034-7167-2024-0540

**Published:** 2025-12-15

**Authors:** Suelen Pessata Ferraz, Flavia Giron Camerini, Cintia Silva Fassarella, Danielle Mendonça Henrique, Lucas Rodrigo Garcia de Mello, Vivian Schutz, Juliana Gerhardt Soares Fortunato

**Affiliations:** I Universidade do Estado do Rio de Janeiro. Rio de Janeiro, Rio de Janeiro, Brazil; II University of Central Florida. Orlando, Florida, United States of America

**Keywords:** Patient Safety, Intensive Care Units, Medical Device, Medical Overuse, Critical Care Nursing., Seguridad del Paciente, Unidades de Cuidados Intensivos, Dispositivo Médico, Uso Excesivo de la Medicina, Enfermería de Cuidados Críticos.

## Abstract

**Objectives:**

to describe the occurrence of clinical incidents related to the length of stay of invasive devices in intensive care units.

**Methods:**

this was an observational, cross-sectional, descriptive, and exploratory study involving critically ill patients aged ≥ 18 years who used invasive devices between May 2022 and May 2023. Data were extracted from the Epimed Monitor System^®^ software and analyzed using the R statistical program, version 4.3.1.

**Results:**

a total of 1,766 medical records were analyzed, corresponding to 5,436 devices. Sixty-one incidents involving invasive devices were identified. Mechanical ventilation devices had the highest average duration of use (13.65 days). Healthcare-associated infections related to invasive devices were the most frequent incidents (45.9%).

**Conclusions:**

incidents involving invasive devices in intensive care are associated with the presence of the device. It is recommended to implement strategies to reduce the duration of device exposure and, consequently, the associated incidents.

## RESUMEN

**Objetivos:**

describir la ocurrencia de incidentes clínicos relacionados con el tiempo de permanencia de dispositivos invasivos en unidades de terapia intensiva.

**Métodos:**

estudio observacional, transversal, descriptivo y exploratorio con pacientes críticos, de edad ≥ 18 años, que utilizaron dispositivos invasivos entre mayo de 2022 y mayo de 2023. Los datos fueron extraídos del *software Epimed Monitor System* ^®^ y analizados mediante el programa estadístico R, versión 4.3.1.

**Resultados:**

se analizaron 1.766 historias clínicas, con un total de 5.436 dispositivos. Se identificaron 61 incidentes relacionados con dispositivos invasivos. Los dispositivos de ventilación mecánica presentaron el mayor tiempo medio de permanencia (13,65 días). Las infecciones asociadas a la atención sanitaria vinculadas a dispositivos invasivos fueron los incidentes más frecuentes (45,9%).

**Conclusiones:**

los incidentes relacionados con dispositivos invasivos en terapia intensiva están vinculados a la presencia del propio dispositivo. Se recomienda implementar estrategias que permitan reducir el tiempo de exposición a los dispositivos y, en consecuencia, la ocurrencia de los incidentes asociados.

## INTRODUCTION

Health technologies are essential components for the promotion, prevention, diagnosis, treatment, and rehabilitation of critically ill patients. The increase in life expectancy, especially with improved quality of life, is a positive outcome made possible by technological advances in healthcare^(1)^. However, the overuse of health technologies has often been associated with unsatisfactory outcomes, such as increased healthcare system costs and a higher occurrence of patient-related incidents. A technology is considered overused when its application poses risks that outweigh its benefits, thereby increasing the likelihood of patient incidents^(2)^.

Invasive medical devices are a type of technology frequently used in healthcare, particularly for critically ill patients admitted to intensive care units (ICUs). When used excessively and without individualized criteria, these devices may increase the risk of clinical incidents^(3)^.

According to the World Health Organization (WHO) taxonomy, clinical incidents are “incidents that occur in a healthcare setting caused by clinical procedures that resulted, or could have resulted, in unexpected harm to the patient”^(4)^. In general, such incidents are associated with longer hospital stays^(5)^.

Incidents can have a significant negative impact on patients, especially in low-income countries. It is estimated that the global incidence rate of harmful incidents is 14.2% and 12.7%, totaling 42.7 million incidents with harm worldwide. Approximately 30% of these incidents were associated with patient death^(6)^.

The average duration of device use is an important factor associated with the occurrence of incidents. An integrative review showed the average time until incident occurrence for each device type: orotracheal tube - 7.7 days; indwelling urinary catheter - 8.2 days; central venous catheter - 12 days. The longer the device remains in place, the higher the risk of incident^(7)^.

The prevention of device-related incidents in ICUs depends on the establishment and maintenance of a strong patient safety culture within healthcare institutions. In this regard, incident prevention should be tailored to each institution’s context, aiming to improve the quality of care, enhance safety, and ultimately improve performance indicators^(8)^.

## OBJECTIVES

To describe the occurrence of clinical incidents related to the length of stay of invasive devices in intensive care units.

## METHODS

### Ethical aspects

The study complied with the guidelines of Resolution 466/12 of the Brazilian National Health Council, which regulates research involving human subjects, and was approved on January 2, 2023, under opinion number 5.843.200, CAAE: 65866222.5.0000.5282. The requirement for informed consent was waived, as this was a documentary analysis study.

### Study design, period, and setting

This was a retrospective, cross-sectional, descriptive study. Data were collected using the Epimed Monitor System^®^ between May 2022 and May 2023. Invasive devices were retrospectively assessed in terms of their use in patients, from insertion to removal, noting the occurrence or absence of clinical incidents.

The study followed the 22 items of the Strengthening the Reporting of Observational Studies in Epidemiology (STROBE) checklist^(9)^.

The study was conducted in four ICUs of a university hospital in the city of Rio de Janeiro. The ICUs included general intensive care, cardiac intensive care, general postoperative, and cardiac postoperative units. The hospital has a total of 560 inpatient beds, including 39 adult ICU beds where the study was conducted.

### Population and inclusion/exclusion criteria

The study population consisted of all patients with medical records admitted to the hospital’s ICUs who met the eligibility criteria.

The unit of analysis was the invasive devices. Included were the records of patients aged ≥ 18 years who were admitted to the ICUs of the study hospital between May 2022 and May 2023 and who used one or more of the following devices: intravascular devices (central venous catheter, short-term hemodialysis catheter, arterial catheter, and peripheral venous catheter), invasive mechanical ventilation (via orotracheal tube or tracheostomy), and indwelling urinary catheter. Patients whose devices remained in place for 24 hours or less were excluded, as this short duration did not align with the study’s focus on prolonged device use. For incident analysis, losses included duplicates (records entered more than once into the Epimed Monitor System^®^) and records with incomplete data.

### Study Protocol

The study was conducted using the Epimed Monitor System^®^, which is integrated with the hospital’s electronic medical record system - MV Soul^®^. Epimed is a commercial, reliable, and secure cloud-based software designed to support quality improvement and benchmarking in hospitals that adopt it. This integration allows for the automatic import of patients’ demographic and clinical data into the system. Additional information is regularly entered into the software by trained hospital staff based on entries in the electronic medical record and direct observation *in loco*.

The Epimed program includes various input and calculated variables. In this study, demographic variables and clinical information related to severity, frailty, and patient mortality were extracted using specific indicators. The Simplified Acute Physiology Score 3 (SAPS 3) was used as a severity indicator and hospital mortality predictor, based on information collected within the first hour of ICU admission. Patient frailty was assessed based on cognitive capacity, functional ability, and pre-existing conditions at the time of admission, as represented by the Modified Frailty Index (MFI).

Another variable extracted was the Standardized Mortality Ratio (SMR), calculated as the ratio between actual ICU mortality and the predicted average mortality, as estimated by a disease severity score (SAPS 3). An SMR < 1 indicates good ICU performance, whereas values > 1 suggest poor performance^(10)^.

Benchmarking is one of the tools available within the Epimed program and is used for quality management in healthcare institutions. It consists of evaluating a facility in comparison with its peers. The main objective of this tool is to highlight differences and similarities in service performance. Accordingly, national and international hospitals using the Epimed Monitor System^®^ can benchmark their performance against local, regional, national, and even international standards.

In this study, benchmarking was conducted by comparing the ICUs of the hospital where the study took place with other ICUs from hospitals also using the Epimed Monitor System^®^. The ICU types included in this benchmarking analysis were: public hospital ICUs, private hospital ICUs, ICUs from internationally accredited hospitals, and Top Performer ICUs. The purpose of benchmarking the studied hospital against other institutions was to compare differences and similarities in best practices observed in real-world settings.

Public hospital ICUs are funded and maintained by the government and serve patients under the Unified Health System (SUS in Portuguese). Private hospitals are funded either by patients themselves or through private health insurance plans. Accredited hospitals are those that have undergone an evaluation and certification process based on predefined standards and criteria to promote quality and safety. Once approved, they receive certification with a quality seal. Top Performer ICUs are those that achieve the best clinical outcomes with efficient resource allocation in the care of their patients. This certification is part of a project created by Epimed Solutions^®^ in collaboration with the Brazilian Intensive Care Medicine Association (AMIB).

Devices were categorized by type and duration of use. Clinical incidents related to devices were characterized by type, associated device, event occurrence, and duration of use at the time of the event, as well as incident severity (i.e., harm caused to the patient), classified as: mild (minimal harm), moderate (harm or long-term/permanent loss of function with extended hospitalization), and severe (harm resulting in reduced life expectancy with extensive permanent loss of function)^(11)^. Incident severity classification is available within the program and was entered by the institution’s professionals at the time of incident reporting.

### Data Analysis and Statistics

Data were exported from the software to Microsoft Excel^®^ 2019, where they were organized and later processed and analyzed with the assistance of a professional statistician. Descriptive statistical techniques were used, and results were expressed as means and frequencies using the R statistical package, version 4.3.1^(12)^.

## RESULTS

From the Epimed Monitor System^®^ software, 6,791 devices were identified as having been inserted in 2,167 patients, with 350 incidents recorded. After applying the eligibility criteria, 1,766 patients were included in the study, with 5,436 devices and 61 incidents documented. This was a descriptive study; therefore, risk analyses or predictive models were not conducted for this data subset.

The mean age of patients who used the devices was 58.74 years, ranging from 18 to 100 years. Male patients were more prevalent, totaling 955 (54.08%). The average length of ICU stay was 7.3 days, ranging from 1 to 161 days.

The devices included in the study were those most frequently used and readily available in the ICU. They were categorized as follows: intravascular devices (central venous catheter, peripheral venous catheter, arterial catheter, and hemodialysis catheter); indwelling urinary catheter; and invasive mechanical ventilation devices (orotracheal tubes and tracheostomies). Among the devices included in the study (5,436), 3,871 (71.21%) were intravascular, 1,267 (23.31%) were indwelling urinary catheters, and 298 (5.48%) were mechanical ventilation devices.

Invasive mechanical ventilation had the highest average duration of use, at 13.65 days. The average duration between insertion and removal of the indwelling urinary catheter was 9.66 days. Among the intravascular devices, the hemodialysis catheter had the longest average duration of use, at 13.57 days, as shown in Table 1.

**Table 1:** Characteristics and duration of use of invasive devices inserted in intensive care patients, Rio de Janeiro, Rio de Janeiro, Brazil, 2023 (N = 5,436)

Devices	n	%	Length of stay (days)		
			Mean (days)	SD	CI
Intravascular	3871	71.21	7.30	6.25	7.1 - 7.5
Central venous catheter	1474	38.08	9.06	6.09	8.7 - 9.3
Peripheral venous catheter	1129	29.17	4.09	3.19	3.9 - 4.2
Arterial catheter	1072	27.69	7.12	6.04	6.8 - 7.5
Dialysis catheter	196	5.06	13.57	10.61	12.0 - 15.0
Urinary catheter	1267	23.31	9.66	10.89	9.0 - 10.2
Invasive Mechanical Ventilation	298	5.48	13.65	14.27	12.0 - 15.2

*SD - Standard Deviation; CI - Confidence Interval.*

The main incidents identified were catheter-associated primary bloodstream infection (PBSI) (21.31%), ventilator-associated pneumonia (VAP) (19.67%), and device-related pressure injury (11.48%). Other incidents were recorded at lower frequencies. Regarding severity, 77.35% of the incidents were classified as moderate, as shown in Table 2.

**Table 2:** Classification of clinical incidents related to the use of invasive devices in intensive care units, Rio de Janeiro, Rio de Janeiro, Brazil, 2023 (N = 61)

Variable		n	%
Clinical incident with harm (n = 53)	Primary BSI associated with CVC	13	21.31
	VAP	12	19.67
	Unplanned removal/misplacement of ETT	8	13.12
	MDRPI related to IUC	4	6.55
	Unplanned removal/misplacement of CVC	4	6.55
	UTI associated with IUC	3	4.92
	MDRPI related to CVC	3	4.92
	Phlebitis	1	1.64
	Incident with mechanical ventilator	1	1.64
	Arterial catheter fracture	1	1.64
	CVC obstruction	1	1.64
	IUC obstruction	1	1.64
	Unplanned removal/misplacement of IUC	1	1.64
Clinical incident without harm (n = 8)	Unplanned removal/misplacement of arterial catheter	4	6.55
	Unplanned removal/misplacement of peripheral venous access	3	4.92
	IUC obstruction	1	1.64
Severity of clinical incident with harm (n = 53)	Severe	2	3.77
	Moderate	41	77.35
	Mild	5	9.44
	Not reported	5	9.44

*PBSI - Primary bloodstream infection; CVC - Central venous catheter; VAP - Ventilator-associated pneumonia; ETT - Endotracheal tube; MDRPI - Medical Device-Related Pressure Injury; IUC - Indwelling urinary catheter; UTI - Urinary tract infection; CI - Confidence Interval.*

Regarding the average time between device insertion and the occurrence of the event, the mean duration of mechanical ventilation until the incident was 10 days. The average time between central venous catheter placement and incident occurrence was 9.39 days. The indwelling urinary catheter showed an average duration of 8.4 days until the incident occurred, as shown in Table 3.

**Table 3:** Mean duration, in days, of device use until the occurrence of a clinical incident, Rio de Janeiro, Rio de Janeiro, Brazil, 2023

Device	Mean	SD	Median	CI
Intravascular	8.2	6.6	6.5	6.40 -11.29
Central venous catheter - Short Term	9.4	4.8	9	7.29 - 11.58
Peripheral venous catheter	2.5	1.0	2	1.9 - 3.88
Arterial catheter	5	2.7	5	3.11 - 7.47
Dialysis catheter - Short Term	14.3	16.2	5	3.01 - 35.13
Urinary catheter	8.4	10.0	5	4.32 - 17.87
Invasive Mechanical Ventilation	10.0	12.3	5	6.29 - 17.62

*SD - Standard Deviation; CI - Confidence Interval.*

The benchmarking revealed that patients in the study hospital were more frail (MFI of 2.5) compared to those in other hospitals; however, they were admitted to the ICU with lower severity during the first 24 hours, according to the mean SAPS 3 score. The evaluation of the SMR showed that the study units had poorer performance (1.63) compared to the other hospitals analyzed, as shown in Table 4.

**Table 4:** Benchmarking by hospital type, Rio de Janeiro, Rio de Janeiro, Brazil, 2023

Variables	Study Hospital	Public Hospitals	Private Hospitals	International Accreditation	Top Performer ICUs
Hospitals (n)	1	272	432	80	114
Discharge from unit (%)	85.9	78.43	90.84	93.02	93.82
Unit death (%)	14.1	19.41	7.5	5.51	5.01
Hospital death (%)	20.9	27.58	10.93	8.53	7.65
MFI (points - mean)	2.35	1.45	1.54	1.51	1.7
SAPS 3 (points - mean)	41.48	45.97	42.78	42.64	45.05
SMR	1.63	1.48	0.81	0.66	0.51
(95% CI)	(1.55-1.94)	(1.47-1.5)	(0.8-0.81)	(0.65-0.67)	(0.5-0.52)

*MFI - Modified Frailty Index; SAPS 3 - Simplified Acute Physiology Score 3; SMR - Standardized Mortality Ratio; CI - Confidence Interval.*

## DISCUSSION

The patient profile in this study was characterized by a predominance of male patients, corroborating most of the studies found in the literature^(13,14)^. The average age in Brazilian ICUs indicates a predominance of patients between 50 and 70 years of age^(15)^.

Length of ICU stay is used as an indicator of hospital efficiency, enabling the prediction of bed availability and the evaluation of the effectiveness of care provided in the unit^(16)^.

The average ICU length of stay observed in this study was slightly above the national average for Brazilian ICUs, which was 5.9 days in 2022^(17)^. The National Health Agency establishes a target ICU length of stay for adults between 4.5 and 5.3 days^(18)^.

Length of hospital stay may vary due to complications related to the natural course of the disease, the quality of care, and/or clinical management strategies^(19)^. Therefore, although it is undeniable that progress has been made in the quality of healthcare and treatment in hospital settings, clinical incidents associated with invasive devices remain a frequent challenge, especially in ICUs^(20)^.

The occurrence of incidents may be related to several factors involving healthcare professionals’ practices (such as negligence in care delivery, work overload, lack of knowledge, absence of material and managerial resources, including organizational protocols, among others). However, device dwell time stands out as an important risk factor to be considered in the occurrence of incidents^(19-21)^.

Critically ill patients in intensive care often require invasive devices for therapeutic purposes and life support. The decision to insert invasive devices is guided by the patient’s profile and clinical needs. In this context, the duration of use/exposure to complex technologies designed to prolong survival also exposes these patients to risk factors for the occurrence of incidents^(20)^.

Studies show that the most commonly used devices in ICUs are central vascular catheters ^(20)^. The average length of use of invasive devices in patients who experienced adverse events showed that short-term hemodialysis catheters, invasive mechanical ventilation, and central venous catheters had the longest durations, averaging 14 days, 10 days, and 9 days, respectively. Although assessed individually, the hemodialysis catheter is considered a type of central venous catheter.

When comparing these findings with those of a literature review evaluating the time to incident occurrence, it is evident that patients in this study remained longer with the hemodialysis catheter, mechanical ventilation, and central venous catheter until the incident occurred. The study reinforces that device dwell time is a relevant factor associated with the occurrence of incidents: the longer the device remains in place, the greater the risk of incidents^(5)^.

As a strategy to reduce device dwell time, identifying care-related vulnerabilities and developing plans for prevention and improvement in the quality of care are essential. The development of bundles, checklists, indicators, and even software can support this process. It is also crucial to establish a therapeutic plan that guides patient treatment and allows for the earliest possible device removal. Developing strategies for the prevention of clinical incidents during device insertion and maintenance is of utmost importance.

Device utilization time can be considered a modifiable factor that increases the likelihood of incidents, particularly those of infectious origin. Studies have shown that bloodstream infections associated with central venous catheters increase proportionally with prolonged use. A Brazilian study identified a 2% increase in the risk of incident for each additional day of central venous catheter use^(19-22)^.

The most prevalent device-related incidents in this study were infections, particularly pneumonia and PBSI, associated with invasive mechanical ventilation and short-term central venous catheters, respectively. These findings are consistent with results from national and international studies^(19,20,23-25)^.

Healthcare-associated infections (HAIs) remain the most common incidents affecting critically ill patients, with their incidence in ICUs being substantially higher than in general wards. This is due to the vulnerability of patients who are frequently subjected to invasive procedures^(23-26)^.

HAIs can worsen the hospitalization process and lead to complications in the patient’s health status, which have been linked to longer hospital stays, increased healthcare costs, morbidity, and mortality^(23-27)^.

ICU admission exposes patients to a high risk of pneumonia and other pulmonary complications, especially among those requiring mechanical ventilation^(28)^. International studies report that VAP affects 9% to 28% of ICU patients dependent on invasive mechanical ventilation. In Brazil, the incidence of VAP ranges from 23.2% to 36.01%. This infection has a global mortality rate ranging from 10% to 65%, with an incidence density of 1 to 4 incidents per 1,000 ventilator-days in developed countries and up to 13 incidents per 1,000 ventilator-days in developing countries^(29)^.

The incidence rate of catheter-associated primary bloodstream infection (CVC-PBSI), in general, is high in intensive care settings and has a direct impact on increased hospital stays and additional healthcare costs. In the United States, studies estimate that approximately 30,100 cases of this type of infection occur annually, accounting for 10% of all HAIs^(21-30)^.

Central venous catheter-associated bloodstream infections have a high incidence rate, ranging from 12% to 25%, and contribute to a mortality rate of up to 25%. Latin American countries report significantly higher rates of this type of infection compared to high-income countries^(22)^.

In assessing the severity of incidents in this study, it was found that the majority of resulting harm (85%) was classified as moderate. Moderate harm is defined as permanent or long-term loss of function, with increased length of hospital stay^(11)^.

Brazilian studies that assessed incidents in intensive care have identified that, when incidents occurred, patients generally experienced mild harm. However, a study conducted in the capital of Minas Gerais showed that, when analyzing only incidents with moderate harm, 74% were categorized as HAIs^(19)^. These findings are consistent with those of the present study, in which infections were the most frequently reported incidents, with a predominance of moderate severity classification.

The occurrence of incidents results in prolonged hospital stays, and, simultaneously, longer hospitalization increases the patient’s exposure to new incidents, particularly infections. Studies have demonstrated a significant relationship between incident occurrence and increased mortality, highlighting the severity of complications resulting from healthcare-related harm^(22,31,32)^.

In assessing the health condition of patients in this study, it was observed that they were more frail; however, they were admitted to the ICU with lower severity as predicted by SAPS 3. Although the mortality rate was lower among patients in this study, the SMR was higher (1.6) in the studied ICUs when compared to other public units in Brazil^(17)^.

The evaluation of the severity predictor score (SAPS 3: 41.48), mortality rate, and SMR (1.63) in the studied ICUs revealed results similar to those of public hospital ICUs in Brazil. In 2022, the average SAPS 3 score in these ICUs was 44.11, with a SMR of 1.56^(17)^.

Through comparative benchmarking of Brazilian ICUs (public hospital profile), conducted via the Epimed system during the same study period, it was possible to compare the patient profile data from this study with those of other hospitals. The goal of conducting benchmarking-comparing the studied unit with ICUs from other institutions-is to contrast differences and similarities in best practices observed in real-world settings, thereby fostering improvements in healthcare systems.

It was found that the average patient age and ICU length of stay were similar to those observed in other hospitals. Regarding MFI, patients in this study were considered more frail, although they were admitted to the ICU with lower severity as predicted by SAPS 3. Although the mortality rate was lower among study patients, the SMR was slightly higher (1.6) in the studied ICUs compared to other Brazilian units^(17)^.

It is understood that, although the presence of a device already represents an increased risk factor for incident occurrence, its use is essential for the patient’s life support. Critically ill patients typically require monitoring due to the risk of acute instability, as well as hemodynamic or respiratory support to sustain life. This makes them more susceptible to the use of various invasive devices. Therefore, it is crucial to ensure awareness and daily evaluation of the need for continued use, as well as to implement interventions that minimize complications and allow for the earliest possible device removal^(19-22)^.

Given this, the nurse’s role in the process of incident prevention is fundamental. The presence of this professional at the bedside is indispensable for care delivery, as they are responsible for identifying potential risks, recording and reporting incidents for analysis and the establishment of goals and improvement strategies, managing protocols and training, and most importantly, working daily with the multidisciplinary team to reassess the continued need for these devices.

### Study limitations

Underreporting of incidents remains a limitation in studies of this nature and is linked to the need for a stronger patient safety culture, with encouragement for non-punitive reporting practices. Such an approach facilitates the identification of care vulnerabilities and the development of strategies for improvement.

### Contributions to the Field

Overall, this study made it possible to identify the devices associated with the highest risk of clinical incidents, the most frequent types of incidents, and their impact on complications. As a result, healthcare managers and professionals will be better equipped to target their actions and strategies, promoting higher-quality care and minimizing risks and incidents for patients. Furthermore, benchmarking-by comparing the studied unit with ICUs from other institutions-enabled the identification of differences and similarities in best practices as observed in real-world settings.

## CONCLUSIONS

The most commonly used devices were intravascular catheters, specifically central and peripheral venous catheters. The devices with the longest dwell times were invasive mechanical ventilation devices, followed by hemodialysis venous catheters, indwelling urinary catheters, and central venous catheters.

Among the devices studied, invasive mechanical ventilation was associated with the highest number of incidents, along with central venous catheters and indwelling urinary catheters. Most incidents were classified as moderate in severity. HAIs accounted for the majority of incidents, particularly PBSI and VAP.

Incidents involving invasive devices in intensive care may be influenced by multiple factors and are closely tied to the presence of the device itself. Evaluating the overuse (duration of use) of these devices is a critical step in implementing strategies aimed at reducing exposure time and, consequently, device-related incidents.

The data from this study provide exploratory insights for the development of future investigations aimed at institutional improvement.

## Data Availability

The research data are available in a repository: https://doi.org/10.48331/scielodata.YEV4UK.
