# Supplementary material for: Incidents related to the duration of medical devices in Intensive Care: a cross-sectional study
Source: Rev Bras Enferm. 2025 Dec 15;78(5):e20240540. doi: 10.1590/0034-7167-2024-0540 (PMC12707842; doi:10.1590/0034-7167-2024-0540)
Supplement: Translation

# Translation: Incidentes relacionados ao tempo de dispositivos médicos em Terapia Intensiva: estudo transversal

**DOI:** 10.1590/0034-7167-2024-0540pt

**Authors:** Suelen Pessata Ferraz, Flavia Giron Camerini, Cintia Silva Fassarella, Danielle Mendonça Henrique, Lucas Rodrigo Garcia de Mello, Vivian Schutz, Juliana Gerhardt Soares Fortunato

**Affiliations:** I Universidade do Estado do Rio de Janeiro. Rio de Janeiro, Rio de Janeiro, Brasil; II University of Central Florida. Orlando, Florida, Estados Unidos da América

**Keywords:** Segurança do Paciente, Unidade de Terapia Intensiva, Dispositivo Médico, Sobreutilização da Medicina, Enfermagem de Cuidados Críticos

## Abstract

**Objetivos:**

descrever a ocorrência de incidentes clínicos relacionados ao tempo de permanência de dispositivos invasivos em unidades de terapia intensiva.

**Métodos:**

estudo observacional, transversal, descritivo e exploratório com pacientes críticos, com idade ≥ 18 anos, que utilizaram dispositivos invasivos entre maio de 2022 e maio de 2023. Dados extraídos do *software Epimed Monitor System^®^* e analisados através do programa estatístico *R*, versão 4.3.1.

**Resultados:**

1.766 prontuários, sendo 5.436 dispositivos analisados. Foram identificados 61 incidentes com dispositivos invasivos. Os dispositivos de ventilação mecânica apresentaram maior tempo médio de permanência (13,65 dias). As infecções relacionadas à assistência à saúde associadas a dispositivos invasivos foram os incidentes mais frequentes (45,9%).

**Conclusões:**

os incidentes com dispositivos invasivos na terapia intensiva estão atrelados à presença do dispositivo. Recomenda-se implementar estratégias que possibilitem a redução do tempo de exposição aos dispositivos e, consequentemente, aos incidentes a eles associados.

## INTRODUÇÃO

As tecnologias em saúde são elementos essenciais para a promoção, prevenção, diagnóstico, tratamento e reabilitação de pacientes críticos. O aumento da expectativa de vida, especialmente com qualidade, é um reflexo positivo proporcionado pelo avanço tecnológico na saúde^(1)^. No entanto, a sobreutilização de tecnologias em saúde tem sido frequentemente associada a resultados insatisfatórios, como o aumento de gastos no sistema de saúde e maior ocorrência de incidentes aos pacientes. Uma tecnologia é considerada sobreutilizada quando seu uso expõe a riscos que superam os benefícios e, assim, aumenta as chances de incidentes aos pacientes^(2)^.

Os dispositivos invasivos assistenciais são um tipo de tecnologia utilizada constantemente na assistência à saúde, principalmente em pacientes graves internados em unidade de terapia intensiva (UTI). Quando usados em excesso e sem critérios individualizados, podem aumentar os riscos para ocorrência de incidentes clínicos^(3)^.

Os incidentes clínicos, de acordo com a taxonomia da Organização Mundial da Saúde (OMS), são “incidentes que ocorrem numa unidade de cuidados de saúde provocados por procedimentos clínicos que resultaram, ou podiam ter resultado, em dano inesperado para o doente”^(4)^. Os incidentes, de maneira geral, estão relacionados ao aumento do tempo de internação^(5)^.

Os incidentes podem causar grande impacto negativo para o paciente, principalmente em países com renda baixa. Foi estimada uma taxa de incidência global de incidentes com dano de 14,2% e 12,7%, totalizando 42,7 milhões de incidentes com dano em todo o mundo. Cerca de 30% desses incidentes foram associados à morte do paciente^(6)^.

O tempo de permanência médio do dispositivo é um fator importante relacionado à ocorrência de incidentes. Foi evidenciado, em uma revisão integrativa, o tempo médio de permanência do dispositivo até a ocorrência do incidente, sendo: tubo orotraqueal 7,7 dias; cateter vesical de demora 8,2 dias; cateter venoso central 12 dias. Quanto maior o tempo de permanência dos dispositivos, maior será o risco desses incidentes^(7)^.

A prevenção de incidentes relacionados aos dispositivos nas UTI depende da existência e manutenção de uma cultura positiva na segurança do paciente dentro de instituições de saúde. Nesse sentido, a prevenção de incidentes deve ser pensada de acordo com a realidade de cada instituição, visando contribuir para a melhoria da qualidade assistencial, para maior segurança e, consequentemente, melhoria nos indicadores^(8)^.

## OBJETIVOS

Descrever a ocorrência de incidentes clínicos relacionados ao tempo de permanência de dispositivos invasivos em unidades de terapia intensiva.

## MÉTODOS

### Aspectos éticos

O estudo respeitou as diretrizes da Resolução 466/12 do Conselho Nacional de Saúde, referente a pesquisas que envolvem seres humanos, sendo aprovado em 02 de janeiro de 2023, através do parecer 5.843.200, CAAE: 65866222.5.0000.5282. A aplicação do Termo de Consentimento Livre e Esclarecido foi dispensada por se tratar de um estudo de análise documental.

### Desenho, período e local do estudo

Estudo transversal, descritivo, retrospectivo. Os dados foram obtidos através da coleta dentro do programa *Epimed Monitor System^®^*, no período de maio de 2022 a maio de 2023. Os dispositivos invasivos foram avaliados retrospectivamente quanto à sua utilização nos pacientes, desde o período da inserção até sua retirada, observando a ocorrência, ou não, de incidentes clínicos.

Foram seguidos os 22 itens do *checklist Strengthening the Reporting of Observational Studies in Epidemiology* (*STROBE*)^(9)^.

O estudo foi realizado em quatro UTI de um hospital universitário na cidade do Rio de Janeiro, sendo as unidades: terapia intensiva geral, cardiointensiva, pós-operatório geral e pós-operatório cardíaco. O hospital é composto por 560 leitos de internação, totalizando 39 leitos de UTI adulto em que foi feito o estudo.

### População e critérios de inclusão e exclusão

A população foi composta por todos os pacientes com prontuários internados nos setores de terapia intensiva do hospital em que foi desenvolvido o estudo e que atenderam ao critério de seleção.

A unidade de análise foram os dispositivos invasivos. Foram incluídos os prontuários de pacientes com idade ≥ 18 anos, internados nas terapias intensivas do hospital estudado no período de maio de 2022 a maio de 2023, que fizeram uso de algum dos dispositivos: intravasculares (cateter venoso central e de hemodiálise de curta permanência, cateter arterial e cateter venoso periférico), ventilação mecânica invasiva (por tubo orotraqueal ou traqueostomia) e cateter vesical de demora.

Foram excluídos pacientes que tiveram dispositivo com tempo de permanência igual ou inferior a 24 horas, justificado por ser um tempo reduzido e o estudo relacionar-se ao tempo de uso excessivo dos dispositivos.

Ao analisar os incidentes, foram considerados como perdas e excluídos: duplicatas (inseridos mais de uma vez no programa *Epimed Monitor System^®^*) e os que estavam com dados incompletos.

### Protocolo do estudo

Foi realizada através do programa *Epimed Monitor System^®^*, que faz integração com o prontuário eletrônico do hospital do estudo - *Sistema MV Soul^®^*. O *Epimed* é um *software* comercial, confiável e seguro, fundamentado em nuvem, com finalidade de aprimoramento em qualidade e *benchmarking* dentro dos hospitais que o adquirem. Essa integração permite importar as informações demográficas e clínicas dos pacientes de forma automática para o programa. As informações adicionais são inseridas no *software* regularmente por profissionais do hospital, treinados, através de registro no prontuário eletrônico e observação *in locu*.

Dentro do programa *Epimed* existem diversas variáveis inseridas e calculadas. Neste estudo, foram extraídas variáveis demográficas e informações clínicas de gravidade, fragilidade e mortalidade dos pacientes, por meio de indicadores específicos. O *Simplified Acute Physiology Score 3* (*SAPS-3*) foi utilizado como indicador de gravidade e preditor de mortalidade hospitalar, baseado em informações coletadas dentro da primeira hora de admissão na UTI. A fragilidade dos pacientes foi avaliada a partir de informações sobre capacidade cognitiva, capacidade funcional e doenças preexistentes à internação, sendo retratada pelo *Modified Frailty Index* (*MFI*).

Outra variável extraída foi a *Standardized Mortality Ratio (SMR,* ou Razão de Mortalidade Padronizada), calculada pela relação entre a mortalidade real na UTI e a mortalidade média prevista, obtida por um escore de gravidade da doença (*SAPS-3*). A SMR < 1 é indicadora de bom desempenho da unidade, enquanto valores > 1 predizem mau desempenho da unidade^(10)^.

O *benchmarking* é uma das ferramentas contidas no programa *Epimed* e é utilizada para gestão da qualidade nas instituições de saúde. Consiste em avaliar uma empresa em relação à concorrência. A finalidade desta ferramenta está principalmente em contrastar diferenças e semelhanças nos serviços. Com isso, hospitais nacionais e internacionais que utilizam o *Epimed Monitor System^®^* podem utilizar essa ferramenta para se avaliar em comparação com a concorrência local, regional, nacional e até mesmo internacional.

Neste estudo, o *benchmarking* foi realizado entre as UTI do hospital em que a pesquisa foi desenvolvida, em comparação com outras UTI de instituições hospitalares que também utilizam o sistema *Epimed Monitor System^®^*. Os tipos de UTI utilizados para esse *benchmarking* foram: UTI de hospitais públicos, UTI de hospitais privados, UTI de hospitais com acreditação internacional e UTI *Top Performers*. O objetivo de realizar um *benchmarking*, comparando o hospital estudado com as UTI de outras instituições, é contrastar diferenças e semelhanças nas melhores práticas evidenciadas no mundo real.

UTI de hospitais públicos são aquelas financiadas e mantidas pelo Estado, atendendo pacientes do Sistema Único de Saúde (SUS). Os hospitais privados são os que são financiados pelo próprio paciente ou por seguradoras, através de planos de saúde privados. Os hospitais acreditados são aqueles que passaram por um processo de avaliação e certificação, com padrões e requisitos predefinidos para promover a qualidade e a segurança. Após a aprovação, recebem a certificação com selo de qualidade. As UTI *Top Performers* constituem um tipo de certificação concedida às UTI que obtiveram os melhores resultados clínicos, com alocação eficiente de recursos no cuidado dos pacientes nela internados. Esse é um projeto criado pela *Epimed Solutions^®^* com a Associação de Medicina Intensiva Brasileira (AMIB).

Os dispositivos foram caracterizados por tipo e tempo de permanência. Já os incidentes clínicos associados a dispositivos foram caracterizados com relação ao seu tipo; dispositivo relacionado, ocorrência de evento e tempo de utilização no momento do evento, bem como a severidade do incidente (dano provocado ao paciente), classificada como: leve (danos mínimos), moderada (danos ou perda da função definitiva ou de prazo longo, com aumento do tempo de internação) e grave (danos que acarretam redução da expectativa de vida, com ampla perda de função permanente)^(11)^. A classificação da severidade do incidente é um dado existente no programa, tendo sido inserido pelos profissionais da instituição ao relatar o incidente.

### Análise dos resultados e estatística

Os dados foram exportados do *software* para o programa *Microsoft Excel^®^ 2019*, onde foram organizados para, posteriormente, com auxílio de um profissional estatístico, serem processados e analisados. Foram utilizadas técnicas de estatística descritiva, e os resultados foram expressos como média e frequência, usando o pacote estatístico *R*, versão 4.3.1^(12)^.

## RESULTADOS

A partir do *software Epimed Monitor System^®^*, foram identificados 6.791 dispositivos inseridos em 2.167 pacientes, com ocorrência de 350 incidentes. Após a aplicação dos critérios de elegibilidade, foram incluídos 1.766 pacientes, com 5.436 dispositivos e evidenciados 61 incidentes. Este estudo teve caráter descritivo; sendo assim, análises de riscos ou modelos preditivos não foram realizados nesse recorte dos dados.

A média de idade dos pacientes que utilizaram os dispositivos foi de 58,74 anos, variando entre 18 e 100 anos. O sexo masculino teve a maior frequência, com 955 (54,08%) pacientes. O tempo médio de permanência nas UTI foi de 7,3 dias, com intervalo entre 1 e 161 dias de internação nas unidades.

Os dispositivos incluídos no estudo foram aqueles encontrados com maior frequência e disponibilidade dentro da UTI, sendo divididos em: intravasculares, compreendendo cateter venoso central, cateter venoso periférico, cateter arterial e cateter de hemodiálise; cateter vesical de demora; e dispositivos de ventilação mecânica invasiva, compreendendo tubos orotraqueais e traqueostomias. Entre os dispositivos incluídos no estudo (5.436), foram intravasculares 3.871 (71,21%), cateteres vesicais de demora 1.267 (23,31%) e ventilação mecânica 298 (5,48%).

A ventilação mecânica invasiva foi o dispositivo que apresentou maior tempo médio de permanência, com 13,65 dias. O tempo médio entre a inserção do cateter vesical de demora e sua retirada foi de 9,66 dias. Entre os dispositivos intravasculares, o cateter de diálise foi o que evidenciou o maior tempo de permanência, com uma média de 13,57 dias, conforme Tabela 1.

**Tabela 1:** Caracterização e tempo de permanência de dispositivos invasivos inseridos em pacientes na terapia intensiva, Rio de Janeiro, Rio de Janeiro, Brasil, 2023 (N = 5.436)

Dispositivos	n	%	Tempo de permanência (dias)		
			Média	DP	IC
Intravasculares	3871	71,21	7,30	6,25	7,1 - 7,5
Cateter venoso central	1474	38,08	9,06	6,09	8,7 - 9,3
Cateter venoso periférico	1129	29,17	4,09	3,19	3,9 - 4,2
Cateter arterial	1072	27,69	7,12	6,04	6,8 - 7,5
Cateter de Diálise	196	5,06	13,57	10,61	12,0 - 15,0
Cateter vesical	1267	23,31	9,66	10,89	9,0 - 10,2
Ventilação Mecânica Invasiva	298	5,48	13,65	14,27	12,0 - 15,2

*DP - Desvio Padrão; IC - Intervalo de confiança.*

Os principais incidentes identificados foram infecção primária de corrente sanguínea (ICS) associada ao cateter (21,31%), pneumonia associada à ventilação mecânica (PAVM) (19,67%), lesão por pressão relacionada a dispositivo (11,48%). Outros incidentes foram registrados em menor frequência. No que refere severidade, 77,35% dos incidentes foram classificados como moderados (Tabela 2).

**Tabela 2:** Classificação dos incidentes clínicos relacionados ao uso de dispositivos invasivos em terapias intensivas, Rio de Janeiro, Rio de Janeiro, Brasil, 2023 (N = 61)

Variável		n	%
Incidente clínico com dano (n=53)	ICS primária associada ao CVC	13	21,31
	PAVM	12	19,67
	Retirada não planejada/desposicionamento do TOT	8	13,12
	LP RDM relacionada a CVD	4	6,55
	Retirada não planejada/desposicionamento de CVC	4	6,55
	ITU associada ao CVD	3	4,92
	LP RDM relacionada a CVC	3	4,92
	Flebite	1	1,64
	Incidente com ventilador mecânico	1	1,64
	Fratura de cateter arterial	1	1,64
	Obstrução de CVC	1	1,64
	Obstrução de CVD	1	1,64
	Retirada não planejada/desposicionamento de CVD	1	1,64
Incidente clínico sem danos (n=8)	Retirada não planejada/desposicionamento de cateter arterial	4	6,55
	Retirada não planejada/desposicionamento de acesso venoso periférico	3	4,92
	Obstrução de CVD	1	1,64
Severidade do incidente clínico com dano (n=53)	Grave	2	3,77
	Moderado	41	77,35
	Leve	5	9,44
	Ignorado	5	9,44

*ICS - Infecção de corrente sanguínea; CVC - cateter venoso central; PAVM - Pneumonia associada à ventilação mecânica; TOT - tubo orotraqueal; LP RDM - Lesão por Pressão Relacionada a Dispositivos Médicos; CVD - cateter vesical de demora; ITU - Infecção trato urinário; IC: Intervalo de Confiança.*

Com relação ao tempo médio entre a inserção e a ocorrência do evento, a média de permanência da ventilação mecânica até o acontecimento do incidente foi de 10 dias. Já a média de tempo entre a colocação do cateter venoso central e a ocorrência foi de 9,39 dias. O cateter vesical de demora evidenciou tempo médio de manutenção até o incidente de 8,4 dias (Tabela 3).

**Tabela 3:** Tempo médio, em dias, de permanência do dispositivo até ocorrência do incidente clínico, Rio de Janeiro, Rio de Janeiro, Brasil, 2023

Dispositivo	Média	DP	Mediana	IC
Intravasculares	8,2	6,6	6,5	6,40 -11,29
Cateter venoso central - Curta Permanência	9,4	4,8	9	7,29 - 11,58
Cateter venoso periférico	2,5	1,0	2	1,9 - 3,88
Cateter arterial	5	2,7	5	3,11 - 7,47
Cateter de Diálise - Curta Permanência	14,3	16,2	5	3,01 - 35,13
Cateter vesical	8,4	10,0	5	4,32 - 17,87
Ventilação Mecânica Invasiva	10,0	12,3	5	6,29 - 17,62

*DP - Desvio Padrão; IC - Intervalo de Confiança.*

O *Benchmarking* evidenciou que os pacientes do hospital do estudo eram mais frágeis (MFI de 2,5) quando comparados aos outros hospitais, no entanto internam com menor gravidade nas primeiras 24h de UTI de acordo com a média de pontos do SAPS 3. A avaliação da SMR revelou que as unidades do estudo possuem desempenho inferior (1,63) aos outros hospitais comparados (Tabela 4).

**Tabela 4:** *Benchmarking* por tipo de hospital, Rio de Janeiro, Rio de Janeiro, Brasil, 2023

Variáveis	Hospital do Estudo	Hospitais Públicos	Hospitais Privados	Acreditação Internacional	UTIs Top Performers
Hospitais (n)	1	272	432	80	114
Alta na unidade (%)	85,9	78,43	90,84	93,02	93,82
Óbito na unidade (%)	14,1	19,41	7,5	5,51	5,01
Óbito no hospital (%)	20,9	27,58	10,93	8,53	7,65
MFI (pontos - média)	2,35	1,45	1,54	1,51	1,7
SAPS 3 (pontos - média)	41,48	45,97	42,78	42,64	45,05
SMR	1,63	1,48	0,81	0,66	0,51
(IC 95%)	(1,55-1,94)	(1,47-1,5)	(0,8-0,81)	(0,65-0,67)	(0,5-0,52)

*MFI - Modified Frailty Index; SAPS3 - Simplified Acute Physiology Score 3; SMR - Standardized Mortality Ratio; IC - Intervalo de Confiança.*

## DISCUSSÃO

O perfil dos pacientes neste estudo caracterizou-se pela prevalência do sexo masculino, corroborando a maioria dos estudos encontrados^(13,14)^. A média de idade em UTI brasileiras destaca o predomínio de pacientes com idade entre 50 e 70 anos^(15)^.

O tempo de permanência na UTI é utilizado como indicador de eficiência hospitalar, possibilitando predizer a disponibilidade de novos leitos, além de avaliar a efetividade da assistência prestada na unidade^(16)^.

A média de permanência em UTI dos pacientes avaliados demonstra um tempo um pouco acima da média das UTI brasileiras (5,9 dias) no ano de 2022^(17)^. A Agência Nacional de Saúde estabelece como meta para tempo de internação em UTI adulto entre 4,5 e 5,3 dias^(18)^.

O tempo de internação do paciente pode variar devido a agravos relacionados ao curso natural da doença, ao manejo assistencial e/ou a complicações provenientes da qualidade do cuidado^(19)^. Sendo assim, ainda que não se possa negar a evolução e melhoria na qualidade da assistência e do tratamento à saúde no ambiente hospitalar, os incidentes clínicos associados aos dispositivos invasivos são um desafio que frequentemente acomete os pacientes, principalmente nas terapias intensivas^(20)^.

A ocorrência de incidentes pode estar relacionada a diversos fatores que envolvem a prática dos profissionais de saúde (negligência em relação aos cuidados prestados durante a assistência, sobrecarga de trabalho, falta de conhecimento, ausência de recursos materiais e gerenciais, como protocolos organizacionais, entre outros...). No entanto, destaca-se que o tempo de permanência do dispositivo é um fator de risco importante a ser considerado para a ocorrência dos incidentes^(19-21)^.

Os pacientes críticos internados em terapia intensiva estão propensos à necessidade de dispositivos invasivos com a finalidade de tratamento e manutenção da vida. A decisão pela inserção de dispositivos invasivos é definida pelo perfil do paciente e suas necessidades terapêuticas. Dentro desse contexto, o tempo de utilização/exposição às tecnologias complexas aplicadas para aumentar a sobrevida desses pacientes também os expõe a fatores de risco para a ocorrência dos incidentes^(20)^.

Estudos demonstram que os dispositivos mais utilizados em UTI são os vasculares centrais^(20)^. O tempo médio de uso dos dispositivos invasivos em pacientes que sofreram eventos mostrou que os cateteres de hemodiálise de curta permanência, ventilação mecânica invasiva e cateter venoso central foram os que permaneceram por mais tempo, sendo respectivamente 14 dias, 10 dias e 9 dias. Apesar de ter sido avaliado de forma individual, o cateter de hemodiálise é considerado um tipo de cateter venoso central.

Ao comparar esses dados com resultados evidenciados em uma revisão de literatura que avaliou o tempo de permanência do dispositivo até a ocorrência de incidente, percebe-se que os pacientes deste estudo permaneceram mais tempo com cateter de hemodiálise, com a ventilação mecânica e com cateter venoso central até o incidente. O estudo reforça que o tempo de permanência do dispositivo é um fator que merece destaque quando associado à ocorrência de incidentes: quanto maior o tempo de permanência, maior o risco de incidentes^(5)^.

Como estratégia para reduzir o tempo de permanência do dispositivo, destaca-se a identificação das fragilidades assistenciais e o desenvolvimento de planos para prevenção e melhoria na qualidade do cuidado. A elaboração de *bundles, checklists*, indicadores e até mesmo de *software* auxilia nesse processo. Também é essencial a existência de plano terapêutico que direcione o tratamento do paciente, para assim programar a retirada do dispositivo o mais precocemente possível. O desenvolvimento de estratégias de prevenção de incidentes clínicos na inserção e manutenção dos dispositivos é de suma importância.

O tempo de utilização dos dispositivos pode ser considerado uma condição modificável que aumenta a possibilidade do acontecimento de incidentes, principalmente os de procedência infecciosa. Estudos revelam que infecções de corrente sanguínea associadas ao cateter venoso central aumentam proporcionalmente de acordo com o prolongamento do tempo de uso. Um estudo brasileiro identificou aumento de 2% no risco de incidente para cada dia a mais de uso do cateter venoso central^(19-22)^.

Os incidentes com dispositivos de maior prevalência no estudo foram as infecções, sendo as principais a pneumonia e a ICS, relacionadas aos dispositivos de ventilação mecânica invasiva e ao cateter venoso central de curta permanência, respectivamente. Esses achados corroboram os resultados de estudos nacionais e internacionais^(19,20,23-25)^.

As infecções relacionadas a assistência a saúde (IRAS) ainda são os incidentes mais comuns que acometem pacientes criticamente doentes, e sua incidência em UTI é substancialmente maior do que nas enfermarias, devido à condição de vulnerabilidade dos pacientes, submetidos frequentemente a procedimentos invasivos^(23-26)^.

As IRAS podem agravar o processo de hospitalização e levar a complicações nas condições de saúde do paciente, o que tem sido relacionado ao aumento do tempo de permanência, custos de hospitalização, morbidade e mortalidade^(23-27)^.

A internação em UTI expõe os pacientes a alto risco de pneumonia e outras complicações pulmonares, sobretudo naqueles sob uso de ventilação mecânica^(28)^. Estudos internacionais apontam que a PAVM afeta de 9% a 28% dos pacientes internados em terapia intensiva e dependentes de ventilação mecânica invasiva. No Brasil, a incidência de PAVM varia entre 23,2% e 36,01%. Essa infecção apresenta mortalidade global entre 10% e 65%, com densidade de incidência de 1 a 4 incidentes por 1.000 VM-dia em países desenvolvidos e até 13 incidentes por 1.000 VM-dia em países em desenvolvimento^(29)^.

Já a taxa de incidência de infecção primária da corrente sanguínea relacionada ao uso do cateter venoso central (CVC), de maneira geral, é elevada na terapia intensiva e tem influência direta no aumento do tempo de internação dos pacientes e de custos adicionais. Nos Estados Unidos, estudos estimam que aproximadamente 30.100 casos desse tipo de infecção ocorram por ano, correspondendo a 10% das IRAS^(21-30)^.

As infecções de corrente sanguínea associadas ao cateter venoso central apresentam taxa de incidência elevada, entre 12% e 25%, contribuindo com uma mortalidade de até 25%. Países latino-americanos apresentam taxas expressivamente mais elevadas desse tipo de infecção em comparação com países de renda mais alta^(22)^.

Ao avaliar a severidade dos incidentes neste estudo, evidenciou-se que os danos gerados foram classificados, em sua maioria (85%), como moderados. Dano moderado é caracterizado por perda da função permanente ou de longo prazo, com aumento do tempo de internação^(11)^.

Estudos brasileiros que avaliaram incidentes em terapia intensiva identificaram que, quando ocorreram, os pacientes sofreram danos leves. No entanto, um estudo realizado na capital de Minas Gerais evidenciou que, ao analisar apenas incidentes com dano moderado, 74% destes eram categorizados como IRAS^(19)^. Esses dados corroboram os achados desta pesquisa, em que os incidentes mais registrados foram infecções, com predominância de classificação de gravidade moderada.

A ocorrência de incidentes acarreta aumento no tempo de permanência hospitalar e, ao mesmo tempo, o elevado tempo de internação expõe o paciente ao acometimento de novos incidentes, principalmente infecções. Estudos demonstraram expressiva relação entre a ocorrência de incidentes e o aumento da mortalidade, evidenciando a gravidade das complicações adquiridas por danos relacionados à assistência à saúde^(22,31,32)^.

Ao avaliar a condição de saúde dos pacientes do estudo, percebe-se que estes eram mais frágeis, porém internaram na UTI com menor gravidade predita pelo *SAPS 3*. Apesar de a taxa de mortalidade ser menor entre os pacientes do estudo, a SMR foi maior (1,6) nas UTI estudadas, quando comparada à de outras unidades públicas brasileiras^(17)^.

A avaliação do índice preditor de gravidade (*SAPS 3:* 41,48), da taxa de mortalidade e da SMR (1,63) das UTI estudadas revela dados similares aos das UTI brasileiras de hospitais públicos. No ano de 2022, a pontuação média do *SAPS 3* nessas UTI foi de 44,11, com SMR de 1,56^(17)^.

Através do *benchmarking* comparativo das UTI brasileiras (perfil: hospitais públicos), realizado pelo sistema *Epimed* para o mesmo período do estudo, foi possível comparar os dados do perfil dos pacientes da pesquisa com os de outros hospitais. O objetivo de realizar um *benchmarking*, comparando a unidade estudada com as UTI de demais instituições, é contrastar diferenças e semelhanças nas melhores práticas evidenciadas no mundo real, estimulando o aprimoramento dos sistemas de saúde.

Constata-se que a média de idade e o tempo de permanência na UTI são similares aos observados em outros hospitais. Com relação ao *MFI*, os pacientes do estudo são considerados mais frágeis, porém internam na UTI com menor gravidade predita pelo *SAPS 3*. Apesar de a taxa de mortalidade ser menor entre os pacientes do estudo, quando se calcula a taxa de mortalidade padronizada, observa-se uma discreta diferença, sendo esta maior (1,6) nas UTI estudadas do que em outras unidades brasileiras^(17)^.

Entende-se que, embora a presença do dispositivo já represente um fator de risco aumentado para ocorrência de incidentes, sua utilização é fundamental para o suporte vital do paciente. O paciente crítico geralmente necessita de monitorização devido ao risco de instabilidade aguda, além de suporte hemodinâmico ou respiratório para manutenção da vida. Isso o torna mais suscetível à utilização de diversos dispositivos invasivos. Todavia, torna-se fundamental o conhecimento e a avaliação diária da necessidade de manutenção de seu uso, bem como a implementação de intervenções que minimizem complicações e possibilitem sua retirada o mais precocemente possível^(19-22)^.

Diante disso, a participação do enfermeiro no processo de prevenção de incidentes é fundamental. A presença deste profissional na vigilância à beira-leito é indispensável para a assistência, identificando possíveis riscos, registrando e divulgando os incidentes para análise e definição de metas e estratégias de melhoria, além de gerenciar protocolos, treinamentos e, principalmente, revisar diariamente junto à equipe multiprofissional a necessidade da permanência desses dispositivos.

### Limitações do estudo

Destaca-se que a subnotificação de incidentes ainda é uma fragilidade para estudos nessa área e está relacionada à necessidade de implementação da cultura de segurança, com incentivo à notificação de forma não punitiva, facilitando a identificação de fragilidades e a formulação de estratégias para melhoria.

### Contribuições para a área

De maneira geral, o presente estudo permitiu identificar os dispositivos com maior risco para incidentes clínicos, além dos incidentes mais frequentes e sua influência nas complicações. Assim, gestores e profissionais assistenciais poderão direcionar suas ações e estratégias, promovendo uma assistência de qualidade e minimizando riscos e incidentes aos pacientes. Além disso, o *benchmarking*, ao comparar a unidade estudada com as UTI de demais instituições, possibilitou contrastar diferenças e semelhanças nas melhores práticas evidenciadas no mundo real.

## CONCLUSÕES

Os principais dispositivos utilizados foram os cateteres intravasculares, mais especificamente os venosos centrais e os venosos periféricos. Os dispositivos que apresentaram maior tempo de permanência foram os de ventilação mecânica invasiva, seguidos pelos cateteres venosos de hemodiálise, cateteres vesicais e cateteres venosos centrais.

Dentre os dispositivos estudados, os de ventilação mecânica invasiva foram os que apresentaram maior número de incidentes nas unidades, juntamente com os cateteres venosos centrais e os cateteres vesicais de demora, sendo majoritariamente classificados com severidade moderada. As infecções relacionadas à assistência à saúde somaram o maior número de incidentes, com destaque para ICS e PAVM.

Os incidentes com dispositivos invasivos na terapia intensiva podem ter influência de diversos fatores e estão atrelados à presença do dispositivo. A avaliação da sobreutilização (tempo de utilização) desses dispositivos é uma ação importante para implementar estratégias que possibilitem a redução do tempo de exposição aos dispositivos e, consequentemente, aos incidentes a eles associados.

Os dados deste estudo evidenciam informações exploratórias para o desenvolvimento de investigações futuras, a fim de proporcionar melhorias institucionais.

## Data Availability

Os dados de pesquisa estão disponíveis em repositório: https://doi.org/10.48331/scielodata.YEV4UK.
